# COVID-19 Confinement and Health Risk Behaviors in Spain

**DOI:** 10.3389/fpsyg.2020.01426

**Published:** 2020-06-04

**Authors:** Rubén López-Bueno, Joaquín Calatayud, José Casaña, José A. Casajús, Lee Smith, Mark A. Tully, Lars L. Andersen, Guillermo F. López-Sánchez

**Affiliations:** ^1^Department of Physical Medicine and Nursing, University of Zaragoza, Zaragoza, Spain; ^2^National Research Centre for the Working Environment, Copenhagen, Denmark; ^3^Exercise Intervention for Health Research Group (EXINH-RG), Department of Physiotherapy, University of Valencia, Valencia, Spain; ^4^Faculty of Health Sciences, University of Zaragoza, Zaragoza, Spain; ^5^Cambridge Centre for Sport and Exercise Science, Anglia Ruskin University, Cambridge, United Kingdom; ^6^Institute of Mental Health Sciences, School of Health Sciences, Ulster University, Belfast, United Kingdom; ^7^Faculty of Sport Sciences, University of Murcia, Murcia, Spain

**Keywords:** modifiable risk factors, social isolation, Spain, adults, COVID-19

## Abstract

The World Health Organization (WHO) has declared a world pandemic due to COVID-19. In response, most affected countries have enacted measures involving compulsory confinement and restrictions on free movement, which likely influence citizens' lifestyles. This study investigates changes in health risk behaviors (HRBs) with duration of confinement. An online cross-sectional survey served to collect data about the Spanish adult population regarding health behaviors during the first 3 weeks of confinement. A large sample of participants (*N* = 2,741) (51.8% women; mean age 34.2 years [SD 13.0]) from all Spanish regions completed the survey. Binomial logistic regressions adjusted for socioeconomic characteristics (i.e., gender, age, civil status, education, and occupation), body mass index (BMI), previous HRBs, and confinement context (i.e., solitude and exposure to COVID-19) were conducted to investigate associations between the number of weeks confined and a set of six HRBs (physical activity, alcohol consumption, fresh fruit and vegetable consumption, smoking, screen exposure, and sleep hours). When adjusted, we observed significantly lower odds of experiencing a higher number of HRBs than before confinement overall in a time-dependent fashion: OR 0.63; 95% CI: 0.49–0.81 for the second and OR 0.47; 95% CI: 0.36–0.61 for the third week of confinement. These results were equally consistent in all age and gender subgroup analyses. The present study indicates that changes toward a higher number of HRBs than before confinement, as well as the prevalence of each HRB except screen exposure, decreased during the first 3 weeks of COVID-19 confinement, and thus the Spanish adult population may have adapted to the new situational context by gradually improving their health behaviors.

## Introduction

The coronavirus disease 2019 (COVID-19) global pandemic has forced many countries to introduce confinement measures to minimize the propagation of the virus (SARS-CoV-2). This is true for Spain, where the confinement period started on March 15, 2020 (Agencia Estatal Boletín Oficial del Estado, 2020; Gobierno de España, 2020). A period of confinement or quarantine implies a radical change in the lifestyle of the population, disrupting usual daily activities (Jiménez-Pavón et al., 2020). Although quarantine will likely slow the spread of SARS-CoV-2, it may also lead to a higher prevalence of health risk behaviors (HRBs), i.e., behaviors with potentially negative effects on health, such as insufficient physical activity or alcohol consumption above the recommended levels, which may lead to higher levels of anxiety, stress, and depression (Chen et al., 2020; Wang et al., 2020). According to a review conducted by Leppin and Aro (2009), there is no solid theoretical framework for the underlying risk perceptions that may have influenced HRBs in similar pandemics (i.e., SARS and Avian influenza); the majority of studies examining risk perceptions and protective behaviors are not model-based and only preliminary insights are usually provided.

The period of confinement disrupts the usual daily activities of the people that are confined and, in consequence, it is likely that prolonged homestay and solitude will increase sedentary behaviors (sitting, reclining, TV viewing, using mobile devices, or playing videogames) and reduce regular physical activity (Leppin and Aro, 2009; Lin et al., 2018), with a consequently higher risk for cardiovascular disease, cancer, mortality, and poor mental health (Lee et al., 2012; Chekroud et al., 2018; Takagi et al., 2019), and deprivation of acute mitigating effects over stress and mood (Szabo, 2003; Fleming et al., 2020; Wang et al., 2020). Currently, international guidelines recommend at least 150 min per week of physical activity, but it has been suggested that, during the confinement period, physical activity should be increased to at least 200 min per week to compensate for the decrease in the normal daily levels (Jiménez-Pavón et al., 2020). Furthermore, social isolation *per se* is associated with low levels of physical activity and poor diet in a population of young European adults (Hämmig, 2019), although the influence might extend to a wide range of ages since it has also been associated with smoking among older adults (Shankar et al., 2011; Kobayashi and Steptoe, 2018). Also, several studies have linked quarantine to negative psychological effects such as stress, anger, and post-traumatic stress-symptoms (Brooks et al., 2020).

As the COVID-19 epidemic has been found to increase population levels of perceived stress in China, it would be expected that citizens from other COVID-19-afflicted countries would experience a similar increase (Wang et al., 2020). In particular, infection fears, longer quarantine duration, boredom, frustration, inadequate supplies, inadequate information, financial loss, and stigma have been identified as stressors in other quarantine situations; thus, the increase in perceived stress levels could vary in each country depending on the policy adopted regarding the COVID-19 pandemic (Brooks et al., 2020). Moreover, there may be an interplay between COVID-19-related stress and social isolation. Indeed, particular aspects of social isolation, such as social disconnectedness, have been shown to increase the risk of perceived social isolation, which consequently predicted both higher anxiety symptoms and depression symptoms among elderly people (Santini et al., 2020). This could result in exacerbated stress, anxiety, and depression during confinement. Consequently, HRBs closely related to anxiety and stress, such as sleep quality, alcohol consumption, and smoking might be affected during the confinement period (Slopen et al., 2013; Weera and Gilpin, 2019; Xiao et al., 2020a,b). Furthermore, gender, age, and socioeconomic status differences usually lead to different responses as regards stress and HRBs; for instance, current evidence suggests that women are more susceptible to anxiety disorders and tend to smoke more than men to cope with stress (Torres and O'Dell, 2016). Also, the co-occurrence of two or more HRBs has been observed in both adults and older people (Francisco et al., 2019), and higher educational and economic levels seem to inversely correlate with this phenomenon across life (Noble et al., 2015; Mawditt et al., 2016, 2018; John et al., 2018). Similarly, age and gender differences have been pointed to as possible reasons for observed differences among the general population (Mawditt et al., 2016).

In this new situation of COVID-19 confinement, in which general lifestyle is likely to change, there have not yet been any studies analyzing the association between weeks confined due to COVID-19 and HRBs. Therefore, since there is no certainty about when the confinement will finish and how it will influence HRBs, this study aims to analyze the association between time course and HRBs in Spanish adults. This could contribute to informing strategies on how to maintain healthy behaviors among a general population of adults during confinement. Based on previous literature, we hypothesized that a greater length of time in COVID-19 confinement would be associated with unfavorable HRBs.

## Methods

A cross-sectional online survey was conducted to assess associations between time confined and HRBs during the COVID-19 pandemic.

### The Survey

A web-form was used to collect data regarding health behaviors during the period March 22–April 5, 2020 (i.e., from the seventh day of national confinement in Spain being enacted). The survey was launched on social media on March 22, 2020, together with initial information about the objectives of the study. Adults aged 18 years and over currently residing in Spain and self-isolating due to COVID-19 were eligible to participate. Convenience sampling was used to select the participants of the study; according to server analytics, 3,150 media users covering all of the Spanish regions were offered the opportunity to participate. Once they accepted, participants were provided with an information sheet about the study aims and instructions for the survey, gave informed consent to participate, and confirmed whether they were confined. The data provided were anonymous and were treated according to Spanish law regarding general data protection. Once the survey was completed, participants were provided with information regarding health behaviors. The present study retrieved data from 2,741 participants with a mean age 34.2 (SD 13.0) years who completed the survey concerning the following variables: age, gender, civil status, occupation, education, time confined, height, weight, solitude during COVID-19 confinement, exposure to COVID-19, physical activity, screen exposure, sleep time, alcohol consumption, smoking habit, and fresh fruit and vegetable consumption.

### Ethics

The study was conducted following the principles of the World Medical Declaration of Helsinki and was approved by the Ethics Committee of Research in Humans of the University of Valencia (register code 1278789). We reported the study according to the Strengthening the Reporting of Observational Studies in Epidemiology statement (STROBE) (von Elm et al., 2007).

### Time Confined (Exposure)

Participants were asked about the time for which they had been isolated due to mandatory COVID-19 confinement through the following question: “How long have you been isolated due to the COVID-19 confinement enacted?” Possible answers ranged from 1 to 21 days. Participants were later categorized as follows: first week (1–7 days), second week (8–14 days), and third week (15–21 days).

### Health Risk Behaviors (Outcome)

The outcome variable was estimated through a set of questions concerning six health-related behaviors (i.e., exposure to screens, sleep time, physical activity, fruit and vegetable consumption, alcohol consumption, and smoking habit). Participants were asked the following questions: “What is your average daily number of hours exposed to screens such as TV, cell phone, and tablet during COVID-19 confinement?”, with possible answers ranging from “0 h” to “9 or more hours,” “How many hours do you usually sleep a day?”, with answers ranging from “ <5 h” to “more than 9 h,” “How many fresh fruit and vegetables do you usually eat daily?”, with possible answers ranging from “0” to “more than 5,” “Do you usually smoke?”, with possible answers of “current smoker” or “not a current smoker,” and “How often do you drink alcohol?”, with answers comprising “usually,” “moderate,” or “never.” Physical activity was estimated using the Physical Activity Vital Sign (PAVS) short version, in which participants answered two questions regarding the number of days and minutes a week they performed PA, with possible answers comprising 0, 1, 2, 3, 4, 5, 6, or 7 days per week and 10, 20, 30, 40, 50, 60, 90, and 150 or more daily minutes; following the original PAVS procedure, weekly minutes of physical activity were calculated by multiplying days by minutes (Greenwood et al., 2010; Coleman et al., 2012). All of the questions were asked twice to the participants; first, referring to before the confinement status and, second, referring to the confinement status.

We considered HRBs as not achieving the recommendations for each health-related habit. Based on current guidelines and relevant research, each HRB was defined as follows (Table 1): more than 2 h of daily screen time (screen exposure), <6 daily sleep hours (sleep time), less than three fresh fruit or vegetables a day (fresh and vegetable consumption), <150 weekly minutes of moderate to vigorous physical activity (physical activity), any alcohol consumption (alcohol consumption), and a current smoking habit (smoking habit) (World Health Organization, 2010; Grøntved and Hu, 2011; Ma and Li, 2017; Madrid-Valero et al., 2017; Miller et al., 2017; Theodoratou et al., 2017). Participants were categorized into those having a higher number of HRBs than before COVID-19 confinement, and participants having equal or fewer HRBs than before COVID-19 confinement.

**Table 1:** Description of each of the health-risk behaviors included in the study.

**Health-related behavior**	**Description**	**Health risk behavior score**
**Screen exposure**		
	More than 2 h of daily screen time	Yes
	Up to 2 h of daily screen time	No
**Physical activity**		
	<150 weekly minutes of moderate to vigorous physical activity	Yes
	150 weekly minutes of moderate to vigorous physical activity or more	No
**Fresh fruit and vegetable consumption**		
	Less than three fresh fruit or vegetables a day	Yes
	Three or more fresh fruit or vegetables a day	No
**Sleep time**		
	<6 sleep hours daily	Yes
	6 sleep hours or over	No
**Alcohol consumption**		
	Any alcohol consumption	Yes
	No alcohol consumption	No
**Smoking habit**		
	Current smoking habit	Yes
	No current smoking habit	No

### Covariates

According to previous research (Fernandez-Navarro et al., 2018; López-Sánchez et al., 2019), the present study also estimated age, gender, and socioeconomic features (marital status, education, and occupation), as well as self-reported body mass index using World Health Organization (WHO) categories. Moreover, other variables regarding the confinement situation were also controlled: solitude during COVID-19 confinement, and exposure to COVID-19. Self-reported responses were categorized as follows: marital status (“married or having a partner” or “neither married nor having a partner”), education (“having a university degree” or “not having a university degree”), occupation (“employed” or “not employed”), solitude during the COVID-19 confinement (“alone while confined” or “not alone while confined”), and COVID-19 exposure (“infected with COVID-19 or close to an infected person” or “not exposed”). Finally, we also controlled for previous HRBs.

### Statistical Analyses

Statistical analyses were conducted using Stata version 16.1 (StataCorp, Texas, USA). We computed binomial logistic regression tests to check associations between time confined due to COVID-19 and HRBs during the COVID-19 confinement period in Spain, providing odds ratios (ORs) and 95% confidence intervals (CIs) for the whole sample. We also conducted stratified analyses to assess associations concerning gender, and age (i.e., cut-off point of 45 years old, which is a turning point regarding mental health for Spanish men and women) (Ministerio de Salud, 2017), for each and the sum of all HRBs. Participants with missing data in any study variable were discarded for the study (*n* = 143). Levels of significance were set at *p* < 0.05.

## Results

The descriptive statistics of the sample are presented in Table 2. A total of 1,421 participants (51.8%) are women, and 288 (10.5%) declared as being COVID-19-infected or being exposed to someone who was. At the time of questionnaire reply, participants had been confined for an average of 8.8 days (SD 4.4), and 209 (7.6%) were alone while confined. Overall, the number of participants with a higher number of HRBs in comparison with pre-confinement levels while confined was 729 (26.6%).

**Table 2:** Characteristics of the study population and health risk behaviors during COVID-19 confinement.

***N* = 2,741**	***n* (%)**	**Mean (SD)**
Age (y)		34.2 (13.0)
**Gender**		
Men	1,320 (48.2)	
Women	1,421 (51.8)	
**Marital status**		
Married or having a partner	1,216 (44.4)	
Not married or having a partner	1,525 (55.6)	
**Occupation**		
Employed	1,693 (61.8)	
Not employed	1,048 (38.2)	
**Education**		
Holding a university degree	1,680 (61.3)	
Not holding a university degree	1 061 (38.7)	
**Body mass index**		
Underweight	81 (3.0)	
Normal	2,032 (74.1)	
Overweight	437 (15.9)	
Obese	191 (7.0)	
**Alcohol consumption**		
Yes	1,368 (49.9)	
No	1,373 (50.1)	
**Smoking**		
Yes	241 (8.8)	
No	2,500 (91.2)	
**Fruit and vegetable consumption (piece/day)**		
<3	1,383 (50.5)	
≥3	1,358 (49.5)	
**Sleep time (h/day)**		
≤ 6	115 (4.2)	
>6	2,626 (95.8)	
**Screen time (h/day)**		
>2	2,678 (97.7)	
≤ 2	63 (2.3)	
**WHO PA recommendations**		
<150 weekly minutes	1,219 (44.5)	
≥150 weekly minutes	1,522 (55.5)	
**Exposure to COVID-19**		
Yes	288 (10.5)	
No	2,453 (89.5)	
**Alone during COVID-19 confinement**		
Yes	209 (7.6)	
No	2,532 (92.4)	
**Number of previous health risk behaviors**		
0–2	1,314 (47.9)	
3	876 (32.0)	
4–6	551 (20.1)	
**Health risk behaviors during COVID-19 confinement**		
More than before confinement	729 (26.6)	
Equal	1,247 (45.5)	
before confinement	765 (27.9)	
**Week of COVID-19 confinement**		
First	1,591 (58.1)	
Second	615 (22.4)	
Third	535 (19.5)	

**Figure 1:**
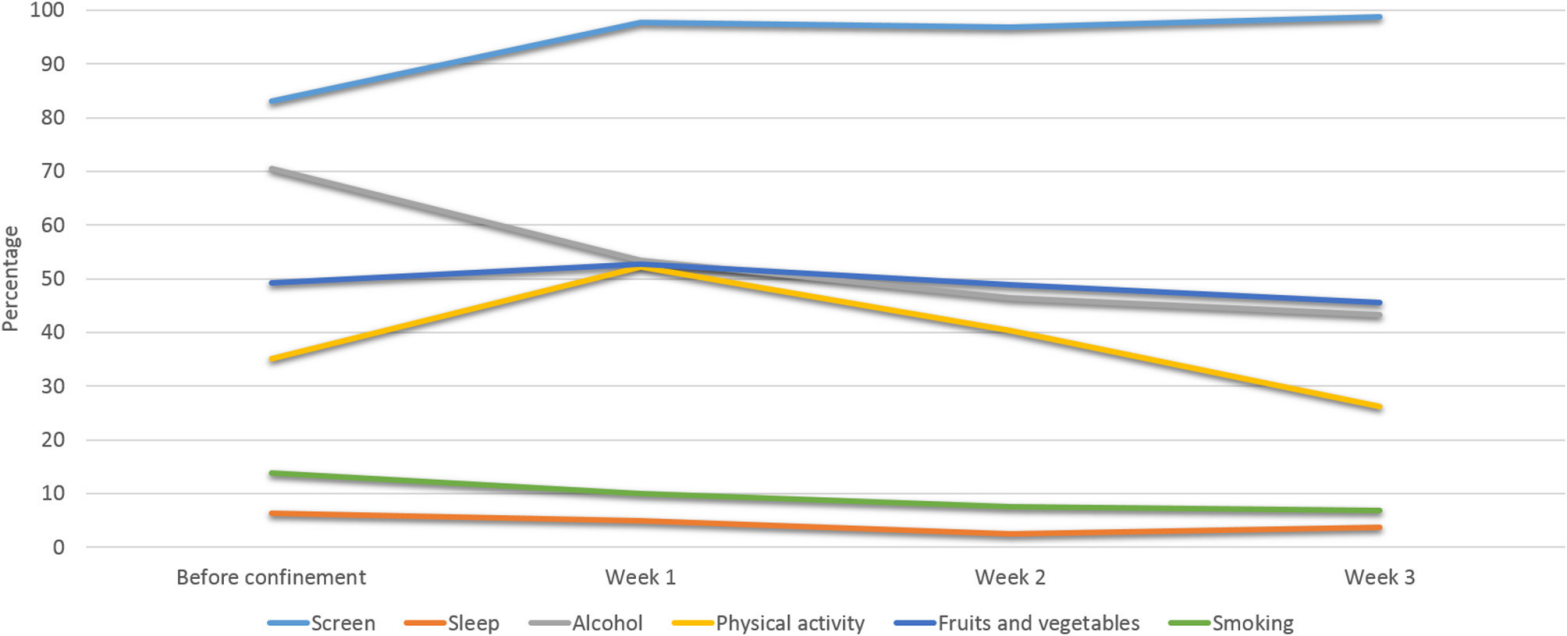
Evolution of percentages of each health risk behavior during the COVID-19 confinement.

As regards specific HRBs, Table 3 and Figure 1 show the evolution of percentages for each HRB (i.e., participants not meeting the recommended guidelines) before and during the COVID-19 confinement period. The percentage of participants meeting the guidelines regarding screen exposure became lower in the course of the confinement period, whereas the percentage of participants meeting the guidelines for the rest of HRBs increased with duration of confinement. Particularly, alcohol consumption and insufficient physical activity prevalence are the two that reduce the most substantially with time-course of confinement. Adjusted logistic regression analyses for each HRB (i.e., not complying with recommended guidelines for each health-related behavior) displayed in Table 4 present significant reduced odds for insufficient physical activity for all participants as well as for all subgroup analyses in a dose-response fashion; overall, fruit and vegetable consumption also show significantly reduced odds for HRB, with the subgroup of participants aged <45 years showing a similar trend.

**Table 3:** Percentage of participants with each health risk behavior previous to and during the COVID-19 confinement.

	**Previous to COVID-19 confinement**		**Week 1**		**Week 2**		**Week 3**		
	***n* (%)**	**Diff. (1–previous)**	***n* (%)**	**Diff. (2–1)**	***n* (%)**	**Diff. (3–2)**	***n* (%)**	**Diff. (3–1)**	P^*^
Screen	2,274 (83.0)	14.7	1,554 (97.7)	−0.8	596 (96.9)	1.8	528 (98.7)	1.0	0.132
Sleep	172 (6.3)	−1.3	80 (5.0)	−2.6	15 (2.4)	1.3	20 (3.7)	−1.3	0.021
Alcohol	1,932 (70.5)	−17.1	850 (53.4)	−6.9	286 (46.5)	−3.2	232 (43.3)	−10.1	<0.001
PA	963 (35.1)	17.1	831 (52.2)	−11.9	248 (40.3)	−14.1	140 (26.2)	−26.0	<0.001
Fruits	1,352 (49.3)	3.5	839 (52.8)	−4.0	300 (48.8)	−3.2	244 (45.6)	−7.2	0.011
Smoke	382 (13.9)	−4.0	157 (9.9)	−2.3	47 (7.6)	−0.7	37 (6.9)	−3.0	0.059

* *Chi-square test among confinement weeks*.

*PA, Physical activity*.

**Table 4:** Adjusted odds ratios (95% confidence interval) for each health risk behavior during COVID-19 confinement in the entire study population and age and gender subgroups (reference group: first week of confinement).

		**Screen exposure**			**Physical activity**			**Fruit and vegetable consumption**		
***N* = 2,741**	**Week**	***n* (%)**	**Model 1^a^**	**Model 2^b^**	***n* (%)**	**Model 1^a^**	**Model 2^b^**	***n* (%)**	**Model 1^a^**	**Model 2^b^**
All	First	1,554 (97.7)	1	1	831 (52.2)	1	1	839 (52.7)	1	1
	Second	596 (96.9)	0.74 (0.42–1.30)	0.75 (0.40–1.39)	248 (40.3)	0.63 (0.52–0.76)	0.61 (0.49–0.76)	300 (48.8)	0.84 (0.70–1.02)	0.77 (0.58–1.01)
	Third	528 (98.7)	1.26 (0.55–2.90)	1.38 (0.58–3.30)	140 (26.2)	0.39 (0.31–0.49)	0.43 (0.33–0.54)	244 (45.6)	0.74 (0.61–0.91)	0.71 (0.53–0.95)
<45 (y)	First	1,132 (98.0)	1	1	541 (46.8)	1	1	613 (53.1)	1	1
	Second	482 (98.2)	1.13 (0.52–2.47)	1.23 (0.53–2.87)	174 (35.4)	0.61 (0.49–0.76)	0.61 (0.47–0.78)	242 (49.3)	0.85 (0.69–1.05)	0.80 (0.59–1.09)
	Third	494 (99.2)	2.33 (0.80–6.80)	2.39 (0.78–7.25)	123 (24.7)	0.38 (0.30–0.49)	0.43 (0.33–0.56)	230 (46.2)	0.77 (0.62–0.95)	0.73 (0.54–0.99)
≥45 (y)	First	422 (96.8)	1	1	290 (66.5)	1	1	226 (51.8)	1	1
	Second	114 (91.4)	0.43 (0.19–1.01)	0.34 (0.13–0.92)	74 (59.7)	0.72 (0.48–1.09)	0.66 (0.42–1.04)	58 (46.8)	0.83 (0.26–1.24)	0.66 (0.34–1.28)
	Third	34 (91.9)	0.34 (0.91–1.27)	0.47 (0.10–2.18)	17 (46.0)	0.43 (0.22–0.85)	0.38 (0.18–0.81)	14 (37.8)	0.56 (0.28–1.12)	0.57 (0.19–1.71)
Men	First	741 (98.9)	1	1	358 (47.8)	1	1	392 (52.3)	1	1
	Second	251 (99.6)	2.73 (0.34–22.04)	3.06 (0.36–26.40)	95 (37.7)	0.71 (0.53–0.96)	0.65 (0.46–0.92)	120 (47.6)	0.83 (0.62–1.11)	0.80 (0.53–1.20)
	Third	314 (98.4)	0.69 (0.22–2.18)	0.93 (0.27–3.21)	56 (17.6)	0.27 (0.19–0.37)	0.29 (0.20–0.42)	140 (43.9)	0.72 (0.55–0.94)	0.71 (0.49–1.04)
Women	First	813 (96.6)	1	1	473 (56.2)	1	1	447 (53.1)	1	1
	Second	345 (95.0)	0.63 (0.34–1.15)	0.59 (0.30–1.17)	153 (42.2)	0.59 (0.46–0.76)	0.59 (0.44–0.78)	180 (49.6)	0.86 (0.67–1.10)	0.74 (0.51–1.08)
	Third	214 (99.1)	2.88 (0.67–12.33)	3.23 (0.72–14.50)	84 (38.9)	0.59 (0.43–0.80)	0.59 (0.42–0.84)	104 (48.2)	0.79 (0.58–1.07)	0.67 (0.42–1.07)
		**Sleep time**			**Alcohol consumption**			**Smoking habit**		
All	First	80 (5.0)	1	1	850 (53.4)	1	1	157 (9.9)	1	1
	Second	15 (2.4)	0.49 (0.28–0.86)	0.40 (0.22–0.74)	286 (43.5)	0.77 (0.64–0.93)	0.89 (0.69–1.16)	47 (7.6)	0.78 (0.55–1.09)	0.99 (0.57–1.72)
	Third	20 (3.7)	0.98 (0.58–1.65)	0.95 (0.54–1.65)	232 (43.3)	0.66 (0.54–0.81)	0.82 (0.63–1.08)	37 (6.9)	0.83 (0.56–1.21)	0.87 (0.47–1.59)
<45 (y)	First	44 (3.8)	1	1	610 (52.8)	1	1	94 (8.1)	1	1
	Second	7 (1.4)	0.35 (0.16–0.79)	0.38 (0.16–0.90)	232 (47.3)	0.93 (0.70–1.25)	0.94 (0.70–1.25)	31 (6.3)	0.75 (0.49–1.14)	0.77 (0.39–1.53)
	Third	17 (3.4)	0.94 (0.53–1.67)	0.98 (0.53–1.82)	211 (42.4)	0.84 (0.63–1.12)	0.81 (0.60–1.08)	32 (6.4)	0.80 (0.52–1.20)	0.74 (0.37–1.46)
≥45 (y)	First	36 (8.3)	1	1	240 (55.1)	1	1	63 (14.5)	1	1
	Second	8 (6.5)	0.73 (0.33–1.62)	0.55 (0.23–1.33)	54 (43.6)	0.87 (0.48–1.56)	0.85 (0.48–1.53)	16 (12.9)	0.82 (0.45–1.84)	1.81 (0.58–5.65)
	Third	3 (8.11)	1.00 (0.29–3.43)	1.00 (0.25–3.96)	21 (56.8)	1.46 (0.48–4.45)	1.49 (0.49–4.55)	5 (13.5)	0.95 (0.36–2.55)	1.20 (0.21–6.98)
Men	First	30 (4.0)	1	1	428 (57.1)	1	1	52 (6.9)	1	1
	Second	5 (1.9)	0.53 (0.20–1.39)	0.49 (0.18–1.40)	120 (47.6)	0.93 (0.64–1.37)	0.93 (0.64–1.37)	19 (7.5)	1.20 (0.66–1.93)	2.10 (0.78–5.66)
	Third	5 (1.6)	0.45 (0.17–1.20)	0.42 (0.15–1.20)	140 (43.9)	1.02 (0.70–1.48)	1.02 (0.70–1.48)	22 (6.9)	1.09 (0.64–1.85)	1.38 (0.56–3.41)
Women	First	50 (5.9)	1	1	422 (50.1)	1	1	105 (12.5)	1	1
	Second	10 (2.8)	0.48 (0.24–0.96)	0.37 (0.18–0.78)	166 (45.7)	0.88 (0.62–1.26)	0.88 (0.62–1.26)	28 (7.7)	0.62 (0.40–0.96)	0.69 (0.34–1.39)
	Third	15 (6.9)	1.53 (0.82–2.85)	1.46 (0.74–2.88)	92 (42.6)	0.62 (0.40–0.94)	0.62 (0.40–0.94)	15 (6.9)	0.63 (0.35–1.18)	0.59 (0.25–1.43)

a *Adjusted for age and gender (all participants), for gender (<45 y, ≥45 y), and for age (men, women)*.

b *Model 1+ socioeconomic features (marital status, occupation, and education), exposure to COVID−19, solitude, body mass index, and previous health risk behavior*.

Overall, participants experiencing their second and third week of confinement, respectively, show significant lower odds for a higher number of HRBs (i.e., healthier lifestyles) in model 1 (Table 5) (OR 0.63; 95% CI: 0.51–0.79) (OR 0.65; 95% CI: 0.51–0.83) than those experiencing 1 week of confinement; even when fully adjusted, participants experiencing 2 and 3 weeks of confinement have progressively and significantly decreased odds for a higher number of HRBs in comparison with pre-confinement levels, with, respectively, OR 0.63; 95% CI: 0.49–0.81 and OR 0.47; 95% CI: 0.36–0.61. Table 5 also shows age and gender subgroup analyses, which display similar significant trends as for the adjusted overall group. Crude analyses for older participants and women in their third week of confinement show no significant association with a higher number of HRBs; when adjusted, both subgroups present significant associations, with, respectively, OR 0.44; 95% CI: 0.20–0.99 and OR 0.55; 95% CI: 0.36–0.83.

**Table 5:** Adjusted odds ratios (95% confidence interval) for a higher number of health risk behaviors than before COVID−19 confinement in the entire study population and age and gender subgroups (reference group: first week of confinement).

***N* = 2,741**	**Week**	***n* (%)**	**Model 1^a^**	**Model 2^b^**
All	First	1,591 (58.1)	1	1
	Second	615 (22.4)	0.63 (0.51–0.79)	0.63 (0.49–0.81)
	Third	535 (19.5)	0.65 (0.51–0.83)	0.47 (0.36–0.61)
<45 (y)	First	1,155 (53.9)	1	1
	Second	491 (22.9)	0.64 (0.50–0.83)	0.69 (0.51–0.92)
	Third	498 (23.2)	0.64 (0.50–0.83)	0.48 (0.36–0.64)
≥45 (y)	First	436 (73.0)	1	1
	Second	124 (20.8)	0.60 (0.38–0.94)	0.52 (0.32–0.86)
	Third	37 (6.2)	0.73 (0.35–1.52)	0.44 (0.20–0.99)
Men	First	749 (56.7)	1	1
	Second	252 (19.1)	0.72 (0.52–1.00)	0.67 (0.46–0.97)
	Third	319 (24.2)	0.60 (0.44–0.83)	0.41 (0.28–0.54)
Women	First	842 (59.3)	1	1
	Second	363 (25.6)	0.57 (0.42–0.77)	0.60 (0.42–0.84)
	Third	216 (15.2)	0.73 (0.51–1.05)	0.55 (0.36–0.83)

a *Adjusted for age and gender (all participants), for gender (<45 y, ≥45 y), and for age (men, women)*.

b *Model 1+ socioeconomic features (marital status, occupation, and education), exposure to COVID-19, solitude, body mass index, and previous health risk behaviors*.

## Discussion

Our study provides novel data from an unusual setting of free movement restrictions resulting from the COVID-19 pandemic. The most critical finding of this study in a large sample of the Spanish adult population was that the odds of having a higher level of HRB (i.e., a change toward a higher number of HRBs than before the confinement) decreased during the confinement due to COVID-19. Contrary to our hypothesis, the prevalence of HRBs improved with longer confinement (i.e., physical activity and consumption of fruit and vegetable increased, tobacco and alcohol consumption decreased, and sleep quality improved), except for screen exposure time. Thus, the population gradually adapted their health behavior with time but also spent more time exposed to screens.

In the case of physical activity, the percentage of people doing <150 weekly minutes increased the first week of confinement but decreased the second and third week. This phenomenon might have occurred because the first week of confinement was used to adjust usual routines to the new context and, thereafter, home-based physical activity started to increase. This result agrees with previous research that found home-based physical activity to have a considerably better adherence (long-term maintenance) rate than center-based physical activity (Ashworth et al., 2005); interestingly, these values for HRB as regards physical activity gradually decreased whereas prevalence for screen exposure HRB remained very high. This point deserves a closer look and further investigation, since higher amounts of sedentary behavior, measured largely as screen time, have been usually associated with lower physical activity levels (O'Donoghue et al., 2016),

Regarding screen exposure, the percentage of participants dedicating more than 2 h to screen exposure daily slightly increased. This is an expected result due to the promotion of both remote work and online education during the COVID-19 confinement (Agencia Estatal Boletín Oficial del Estado, 2020). The high values found in this study for daily screen time far exceed the recommended levels for adults, which could contribute to the experience of mental health disorders such as depression (Wang et al., 2019).

Concerning alcohol and tobacco, the consumption of both decreased during the course of confinement. It seems that during this period, in which health is even more important than usual, people may be trying to adopt healthier lifestyles. Nevertheless, the values found in the present study were, respectively, higher and lower for alcohol and cigarette consumption when compared with prior research involving Spanish participants; such different percentages could be due to differences regarding sample characteristics (e.g., an overall different age may lead to different healthy habits) as well as assessment tools (e.g., alcohol consumption threshold was considered differently in the studies) (Peacock et al., 2018). The increasing use of new technologies in leisure time as substitutes for alcohol and tobacco consumption might be a possible explanation for this reduction trend (Gil-Madrona et al., 2019). Furthermore, longer confinement periods might show different results due to increased stress, especially in very specific populations (e.g., those with impulsive behaviors and/or ex-addicted) (Clay and Parker, 2020), as well as in women (Torres and O'Dell, 2016); this may result from either limited access to supplies or attempts to preserve supplies during the confinement; also, the deprivation of physical social interactions might mitigate both alcohol consumption and smoking (Knudsen et al., 2007; Seid, 2016). Further research would be required to better understand these points.

The percentage of people sleeping for fewer than 6 daily hours per day decreased during the confinement. This is likely to have happened because, during confinement, people do not need to awaken as early to commute to work or may have less job stress. Both job stress and work overload have been associated with poor sleep quality (Shiffman et al., 2009). However, this might especially occur among those with increased social capital, as has recently been shown during the COVID-19 virus epidemic in central China (Xiao et al., 2020a). Also, it is likely that achieving the weekly recommended amount of physical activity or maintaining the usual meal times helped in improving sleep quality (Potter et al., 2016; Altena et al., 2020). Besides, the fact that the HRB regarding sleep time is very low in this study may indicate a moderating influence over the higher anxiety levels associated with the COVID-19 pandemic (i.e., lower sleep deprivation during the confinement might lead to lower anxiety levels) (Pires et al., 2016; Nollet et al., 2020).

Concerning fruit and vegetable consumption, the percentage of people eating fewer than three fresh fruit or vegetables a day decreased during the confinement. This positive result agrees with the food and nutrition recommendations for the Spanish population during the COVID-19 health crisis and could be related to the fact that forced confinement and closure of both bars and restaurants might lead to consuming more home-made cooking (Academia Española de Nutrición y Dietética, 2020). Furthermore, the general tendency toward healthier behaviors as a whole observed in this study might be partially explained by the positively interrelated behavioral domains observed in prior research (i.e., individuals would have decided to lead a healthy lifestyle overall instead of placing emphasis on a single health behavior); in particular, a higher amount of physical activity has been observed to correlate with higher fruit and vegetable consumption (Fleig et al., 2015).

Regarding the influence of the control variables over the association between weeks of confinement and health risk behaviors, this study found a consistent influence of occupation and exposure to COVID-19 (i.e., those participants employed or exposed to COVID-19 had significantly higher odds for HRBs) (results not published). Thus, those working more hours might have less time to take care of their health (leisure-time physical activity, preparing healthier food, sleeping more) and be more exposed to screens due to remote work. This health-related behavior pattern is consistent with findings from previous research, which observed a higher risk of suffering from coronary heart disease and stroke with long working hours (Kivimäki et al., 2015). Furthermore, socioeconomic features may probably explain a substantial part of the differences found among gender subgroups; for instance, women and the higher educated have shown healthier behaviors regarding diet, whereas higher income has been identified as a predictor of higher levels of physical activity (Garza et al., 2013). Also, cultural differences and the perception styles of individuals have been underscored to be behind the perception of the impact of SARS, which, in turn, might have influenced the ability to deal with HRBs in this new COVID-19 pandemic (Cheng and Tang, 2004). Besides, those individuals living in the most affected countries and most financially affected due to a virus outbreak (i.e., equine influenza) have been suggested to be among the most highly stressed and, thereby, more prone to modifying their HRBs (Taylor et al., 2008). Consequently, future research focused on at-risk populations, such as those with deprived backgrounds or those socially and financially affected by the COVID-19 pandemic, is of special interest; research from a theoretical framework perspective based on either the PEN-3 cultural model or the Triandis model of social behavior could contribute to understanding the social circumstances underlying HRBs in this specific context (Facione, 1993; Iwelunmor et al., 2014).

The strengths of the current study consist of examining a wide and large sample of Spanish adults (i.e., participants representing all the Spanish regions) with a good distribution of males and females, and the analysis of a wide set of variables, including novel variables such as weeks isolated or exposure to COVID-19. Besides, the dose-dependent response remains consistent overall and in subgroup analyses. A key limitation of this study was that data were self-reported, potentially introducing self-reporting and recall bias into the findings. Moreover, since a convenience sampling method was used to recruit participants, there is a possibility of selection bias. Second, due to the observational nature of the study, the results do not allow us to infer any causality. Third, the definition for each HRB was based on both current institutional guidelines and relevant research. However, it should be noted that utilizing different definitions or cut points might lead to different results. Last, because the young population is overrepresented in this study, different results might be obtained with an older sample of participants. The authors recommend that future studies analyze the association between weeks confined due to COVID-19 and changes in health risk behaviors in other countries where the population is confined, in order to check whether the trend found in this study is specific to Spain or is an international trend.

## Conclusion

The results of this study consistently showed that changes toward a number of HRBs in Spanish adults (PA, alcohol, tobacco, sleep time, and consumption of fruit and vegetables) progressively decreased during COVID-19 confinement. The only habit that increased was that of screen exposure time. These results point to a necessity to rethink the current system of work and education and suggest that a progressive adaptation to a system with more remote work and more online education may be beneficial for the improvement of people's health.

## Data Availability Statement

The raw data supporting the conclusions of this article will be made available by the authors, without undue reservation.

## Ethics Statement

The studies involving human participants were reviewed and approved by Ethics Committee of Research in Humans of the University of Valencia. The patients/participants provided their written informed consent to participate in this study.

## Author Contributions

RL-B, GL-S, JAC, LS, and JCal contributed the conception and design of the study. RL-B organized the database. RL-B and GL-S performed the statistical analysis. RL-B and GL-S wrote the first draft of the manuscript. JCal, LA, JCas, LS, LA, MT, and JAC wrote sections of the manuscript. All authors contributed to manuscript revision and read and approved the submitted version.

## Conflict of Interest

The authors declare that the research was conducted in the absence of any commercial or financial relationships that could be construed as a potential conflict of interest.
