# Corrigendum: COVID-19 Confinement and Health Risk Behaviors in Spain

**DOI:** 10.3389/fpsyg.2021.789989

**Published:** 2021-11-04

**Authors:** Rubén López-Bueno, Joaquín Calatayud, José Casaña, José A. Casajús, Lee Smith, Mark A. Tully, Lars L. Andersen, Guillermo F. López-Sánchez

**Affiliations:** ^1^Department of Physical Medicine and Nursing, University of Zaragoza, Zaragoza, Spain; ^2^National Research Centre for the Working Environment, Copenhagen, Denmark; ^3^Exercise Intervention for Health Research Group (EXINH-RG), Department of Physiotherapy, University of Valencia, Valencia, Spain; ^4^Faculty of Health Sciences, University of Zaragoza, Zaragoza, Spain; ^5^Cambridge Centre for Sport and Exercise Science, Anglia Ruskin University, Cambridge, United Kingdom; ^6^Institute of Mental Health Sciences, School of Health Sciences, Ulster University, Belfast, United Kingdom; ^7^Faculty of Sport Sciences, University of Murcia, Murcia, Spain

**Keywords:** modifiable risk factors, social isolation, Spain, adults, COVID-19

In the original article, the reference for Chen et al. (2009) was incorrectly written as “Chen, P., Mao, L., Nassis, G. P., Harmer, P., Ainsworth, B. E., and Li, F. (2009). Wuhan coronavirus (2019-nCoV): the need to maintain regular physical activity while taking precautions. *J. Sport Health Sci*. 9, 103–104. doi: 10.1016/j.jshs.2020.02.001”. It should be “Chen, P., Mao, L., Nassis, G. P., Harmer, P., Ainsworth, B. E., and Li, F. (2020). Coronavirus disease (COVID-19): The need to maintain regular physical activity while taking precautions. *J. Sport Health Sci*. 9, 103–104. doi: 10.1016/j.jshs.2020.02.001”.

The authors apologize for this error and state that this does not change the scientific conclusions of the article in any way. The original article has been updated.

## Publisher's Note

All claims expressed in this article are solely those of the authors and do not necessarily represent those of their affiliated organizations, or those of the publisher, the editors and the reviewers. Any product that may be evaluated in this article, or claim that may be made by its manufacturer, is not guaranteed or endorsed by the publisher.
